# Interventions for the endodontic management of non-vital traumatised immature permanent anterior teeth in children and adolescents: a systematic review of the evidence and guidelines of the European Academy of Paediatric Dentistry

**DOI:** 10.1007/s40368-017-0289-5

**Published:** 2017-05-15

**Authors:** M. Duggal, H. J. Tong, M. Al-Ansary, W. Twati, P. F. Day, H. Nazzal

**Affiliations:** 1 Department of Paediatric Dentistry, Leeds Dental Institute, University of Leeds, The Worsley Building, Clarendon Way, Leeds, LS2 9LU UK; 2 Discipline of Orthodontics and Paediatric Dentistry, Faculty of Dentistry, National University of Singapore, 11 Lower Kent Ridge Road, Singapore, 119083 Singapore

**Keywords:** Calcium Hydroxide apexification, Endodontics, Immature non vital incisors, Mineral Trioxide aggregate, Regeneration/revitalisation, Trauamtized teeth

## Abstract

**Aim:**

This systematic review was undertaken in order to develop guidelines for the European Academy of Paediatric Dentistry for the management of non-vital permanent anterior teeth with incomplete root development.

**Methods:**

Three techniques were considered; apexification by single or multiple applications of calcium hydroxide, use of Mineral Trioxide Aggregate (MTA) for the creation of an apical plug followed by obturation of the root canal, and finally a Regenerative Endodontic Technique (RET). Scottish Intercollegiate Guideline Network (SIGN) Guidelines (2008) were used for the synthesis of evidence and grade of recommendation.

**Results:**

Variable levels of evidence were found and generally evidence related to these areas was found to be weak and of low quality. It was not possible to produce evidence-based guidelines based on the strength of evidence that is currently available for the management of non-vital immature permanent incisors.

**Conclusions:**

Based on the available evidence the European Academy of Paediatric Dentistry proposes Good Clinical Practice Points as a guideline for the management of such teeth. It is proposed that the long term use of calcium hydroxide in the root canals of immature teeth should be avoided and apexification with calcium hydroxide is no longer advocated. The evidence related to the use of a Regenerative Endodontic Technique is currently extremely weak and therefore this technique should only be used in very limited situations where the prognosis with other techniques is deemed to be extremely poor. The current review supports the use of MTA followed by root canal obturation as the treatment of choice.

## Introduction

Dental trauma is common in young children and is the most frequent cause of pulpal non-vitality in immature permanent incisors. The 2003 Children’s Dental Health Survey in England and Wales (Harker and Morris 2003) found that 11% of 12 year olds and 13% of 15 year olds had sustained accidental damage to their permanent teeth with the majority of injuries being left untreated. The highest levels of treatment were at age 15 years, of which only 27% had their damaged incisors treated.

Managing non-vital immature teeth is extremely challenging due to compromised crown root ratio, thin root dentine walls and wide-open apex lacking an apical stop against which root filling materials can be condensed. Treating these teeth is therefore time consuming and technically difficult. Traditionally the treatment has been aimed at producing a barrier against which a root canal filling material can be placed, thereby preventing the extrusion of material into the surrounding tissues. This has usually and most commonly been achieved through calcium hydroxide (Ca(OH)_2_) apexification technique that involves repeated and prolonged dressing of the root canal. Although this technique has been reliable and with consistent clinical outcomes, there have been recent concerns about the long-term use of Ca(OH)_2_ in root canals. The technique also carries a higher risk of cervical root fractures, with frequencies being related to the stage of root development (Cvek 1992). This is possibly attributed to its hygroscopic and proteolytic properties, which induces desiccation of dentinal proteins and reduces root dentinal wall modulus of elasticity, thus predisposing the tooth to root fracture (Andreasen et al. 2002).

In the last decade the introduction of Mineral Trioxide Aggregate (MTA) has meant that an apical plug can be created by dentists, which allows immediate obturation of the root canal (Pradhan et al. 2006). However, this material remains expensive and also does not confer any qualitative or quantitative increase in root dimensions. In addition, MTA has been shown in an in vitro study to have similar weakening effect on the dentine to that of calcium hydroxide (Twati et al. 2009a).

Recently there has been a paradigm shift in the proposed treatment for such teeth. Uncontrolled longitudinal studies and randomised controlled trials (RCTs) have shown successful continuation of root canal growth following the use of Regenerative Endodontic Therapy (RET). These techniques have been suggested to harness the stem cells present at an apical area of immature incisors, thereby allowing repopulation of the root canal with vital tissues, and allowing continued deposition of hard tissue and further root development (Banchs and Trope 2004).

In order to develop guidelines for the European Academy of Paediatric Dentistry, this systematic review attempts to compare various types of interventions for treating traumatised immature permanent anterior teeth, and their respective effects. This was performed by evaluating the research evidence in these fields using explicit, systematic methods to limit bias (systematic errors) and reduce chance effects, hoping to provide more reliable results upon which conclusions could be drawn and decisions can be made. In drawing conclusions and making recommendations the authors are very mindful of the fact that there are a few randomised controlled trials (RCTs) with low level of bias in these areas which stand up to rigorous scrutiny that is normally applied to such studies.

## Objectives of the review

- To evaluate the relative effectiveness of the following interventions for treating traumatised non-vital immature permanent anterior teeth:
  (a) Apexification techniques;
  (b) Apical plug technique using MTA;
  (c) Regenerative Endodontic Therapy (RET).
- To evaluate any immediate and/or long-term side-effects and limitations of the materials and techniques used.

## Methodology

### Search strategy for identification of studies

A comprehensive search was developed for ensuring that as many studies as possible were identified through a structured electronic search, hand search, and personal contacts.

#### Electronic search strategy

A search for relevant studies began with OVID electronic bibliographic databases using a structured search strategy that was developed by the Trials Search Co-ordinator of the UK Cochrane Oral Health Group to determine an article’s relevance to this review based on the title and abstract.

### Subject search strategy for MEDLINE via OVID

The subject search used a combination of controlled vocabulary and free text terms. There was no restriction on the language of publication.

### Databases searched

The following databases were searched via OVID gateway:

- ACP Journal Club (ACP).
- Cochrane Central Register of Controlled Trials (CENTRAL).
- Cochrane Database of Systematic Reviews (CDSR).
- Cochrane Oral Health Group Trials Register.
- Database of Abstracts of Reviews of Effects (DARE).
- EMBASE databases; the Excerpta Medica Database (EMBASE), EMBASE Drugs and Pharmacology (EMDP), and EMBASE Psychiatry (EMPS).
- Index to Scientific and Technical Proceedings.
- National Library of Medicine-Toxnet (http://toxnet.nlm.nih.gov).
- Ovid MEDLINE(R) (1966 to date).
- Ovid MEDLINE(R) (daily update).
- Ovid MEDLINE(R) in-process, other non-indexed citations.
- Science Citation Index Expanded.
- Social Science Citation Index.
- System for Information on Grey Literature in Europe.

#### Personal contacts

Personal communication via e-mail correspondence was initiated with the author(s) of the identified relevant studies in an attempt to identify on-going, unpublished or unlisted studies that may be eligible for inclusion in this review. A list of these relevant articles along with the inclusion criteria for the review was sent to the first author of those reports concerning the studies included asking if they knew of any additional studies (published or unpublished) that might be relevant. The same correspondence was sent to other experts and others with an interest in the area.

The manufacturers of dental materials were contacted to obtain information on relevant published or unpublished studies that may have involved the materials that they manufacture. Companies were also asked for reference lists that contain studies on dental materials that are used in inducing a calcific barrier and root strengthening procedures.

### Study selection process

All reports identified electronically were scanned on the basis of the title, keywords and abstract to exclude reports that were non-relevant to the review question as well as case reports, in vitro, animal studies, and retrospective studies. In order to ensure that the appraisal criteria were applied consistently, electronically identified trials, appearing to meet the inclusion criteria, were independently reviewed by two calibrated reviewers. Full text articles were obtained from the University of Leeds Health Science Library if the title or the abstract did not provide enough information about the study to make a decision or there was no abstract available.

### Criteria for considering studies

When a controlled clinical trial was identified, the Cochrane methodology assessment for quality of RCTs (Cochrane Collaboration 2011) was used in this systematic review of interventions for non-vital immature teeth. The following describes the criteria that were used for studies considered for inclusion into this review.

#### Types of studies

RCTs on human subjects that assess the effectiveness of one or more methods of inducing an apical barrier in traumatised non-vital immature permanent anterior teeth (since 1966 up to date) with at least 12 months follow-up period.

#### Types of participants

All patients in all age ranges, presenting with non-vital immature open apex permanent anterior teeth as a result of any type of trauma, requiring root canal treatment due to any signs or symptoms related to these teeth. The diagnosis of pulp status in traumatised teeth can be difficult. This has been demonstrated in laser doppler studies, where the pulp has been shown to be healthy but the tooth has not responded to traditional sensibility tests (Gazelles et al. 1988). The diagnosis of non-vital immature teeth should depend on trauma history (including previous episodes of trauma) as well as one or more clinical signs and symptoms (abscess formation, sinus tracts), negative sensibility testing or radiographic evidence of arrested root development or pulp necrosis.

All patients presenting for root canal obturation following any of the procedures used for inducing an apical barrier in traumatised non-vital immature permanent anterior teeth.

#### Types of interventions

All techniques for inducing an apical barrier in traumatised non-vital immature permanent anterior teeth, including apexification, apical plug formation using MTA, and RET were included. In addition, studies using conventional root canal obturation with no induction of an apical barrier were also included.

#### Types of outcome measures

The main outcome measures were long-term success (asymptomatic with clinical and radiographic signs of healing).

For comparison of different methods of inducing an apical barrier, apexification was compared to apical plug techniques and both techniques were compared to no treatment (conventional root canal obturation or no treatment at all). The primary outcome measure was the proportion of teeth that were symptom-free for at least 12 months after treatment.

The secondary outcome measure was the total duration of treatment time to achieve an apical barrier.

### Assessment of methodological quality of selected trials

The methodological quality of included RCT studies was assessed using the criteria described in the Cochrane Handbook for Systematic Reviews of Interventions 4.2.8. (Cochrane Collaboration 2011) Two reviewers assessed the included trials independently for quality and in duplicate without blinding the name of authors, institutions or journals. The grading for the recommendations in evidence considering all usable studies was performed according to the Scottish Intercollegiate Guidelines Network (SIGN) guidelines (2008).

### Data collection

A data extraction proforma was developed, agreed and tested at the start of data collection stage. The following was included:

- The year of publication and country of origin.
- Sample size, and drop outs/withdrawals.
- Study participants demographics and outcomes measures.
- Detailed description of interventions, techniques and materials used.
- Signs and symptoms before intervention and after treatment.
- Duration of studies.
- Information on adverse events or effects as well as cost implications.

## Results and discussion

### Apexification

Completed searches from all sources identified 200 reports on apexification. Following scanning of the titles and abstracts of these reports; 33 electronically identified reports were not relevant to the review topic and were rejected leaving 167 reports of different study designs to be assessed. The abstracts and full text were obtained whenever there was a doubt that the article could not be definitely rejected. Only six studies were suitable to be assessed as clinical trials and these were assessed in detail, are presented in Table 1.

**Table 1:** Characteristics of controlled calcium hydroxide apexification clinical studies

Author	Level of evidence	n (Teeth)	Drop out	Age years	Length of follow-up	Intervention	Outcome Success
(Roberts and Brilliant 1975)	1-	Exp = 8 Cont = 8	1	Reported for each patient	No follow up after obturation	Ca(OH)_2_ and TCP	High success numbers too small for conclusion
(Mackie et al. 1994)	2	Exp = 19 Cont = 19	3	6–10 11 & older	No follow up after apical closure	Two Ca(OH)_2_ pastes	100%
(Coviello and Brilliant 1979)	1-	Exp = 42 Cont = 59	14	Not defined	9 months	Ca(OH)_2_ + TCP & Ca(OH)_2_ Apexification	82% with Ca(OH)_2_ + TCP & 63.5 with Ca(OH)_2_ Apexification
(Yates 1988)	1-	Exp = 22 Cont = 26	NR	Mean 10.3 9.5	1–7 years	Ca(OH)_2_ Apexification	100% with 9 months better formation
(Merglova 2001)	1-	Exp = 103 Cont = 193	NR	6–15	1–4 years	Ca(OH)_2_ Apexification	94.2%
(Dominguez Reyes et al. 2005)	1-	Exp = 26 Cont = 13	1	6–9	No follow up after obturation	Ca(OH)_2_ Apexification	100%

*NR* Not reported; *TCP* Tricalcium phosphate

Out of those six studies three (Roberts and Brilliant 1975; Coviello and Brilliant 1979; Mackie et al. 1994), met most of the review’s methodological quality assessment criteria. The results reported by Roberts and Brilliant (1975) showed 87.5% (7 out of 8 teeth) successful apical barrier formation using Ca(OH)_2_ powder compared to 75% (6 out of 8 teeth) treated with tricalcium phosphate (TCP). The small numbers of participants in both groups did not allow identification of any difference between materials. One case in the Ca(OH)_2_ group dropped out which would have reduced the success rate to 75% in this group if an intention to treat (ITT) analysis had been performed.

Coviello and Brilliant (1979) reported success in apical barrier formation of 82.9% (29 teeth out of 35) in the apical plug group which had one failure, seven drop-outs and five questionable teeth. The calculated ITT = 69% success. In the apexification group there were nine failures, seven cases dropped out and 10 questionable teeth with a success of 63.5%. The calculated ITT = 55.9% success. There was no reported significant difference between treatment groups or materials used employing Chi-square tests at p<0.05 probability. The relative effectiveness of the single appointment technique using both materials compared to the multi-appointment technique using the same materials cannot be evaluated based on the data presented in this study. The difference in providing treatment to both groups may explain the large number of failures seen in the multi-appointment group which denotes a high risk of performance bias. The same conclusion can be applied to the number of visits needed to complete any treatment in this group. The small numbers of the positive controls in both groups would not allow for identifying any difference between both materials using either technique.

Mackie et al. (1994) compared two Ca(OH)_2_ (Reogan Rapid to Hypo-cal) paste preparations. The success for both brands was 100% based on available patients at the time of final analysis (33 children with 38 teeth out of 36 children with 41 teeth). Cases that dropped out (1 patient with 1 tooth) and excluded cases (2 patients with 2 teeth) were not included in the final analysis. ITT analysis if completed would change the total success rate into 92.7%, which would still be a favourable outcome. It should be noted that both comparison groups were Ca(OH)_2_ preparations and the results should be interpreted on the basis of comparing the two pastes and not to be applied as a general success rate for Ca(OH)_2_ material in multi-visit apexification.

**Fig. 1:**
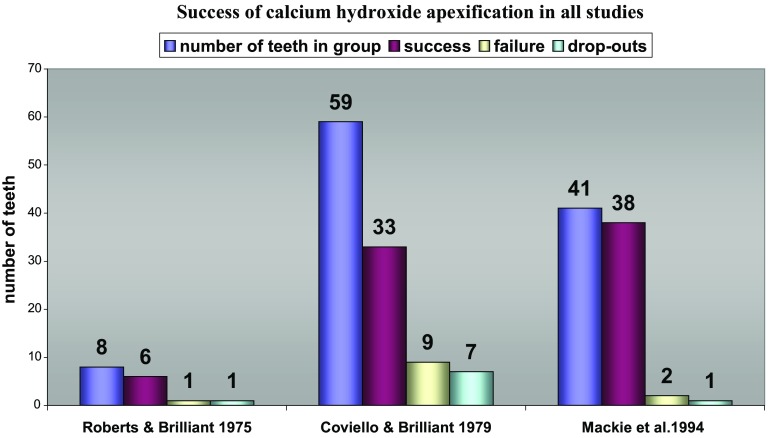
Success of calcium hydroxide apexification in three studies

The overall success rates reported in these studies is summarised in Fig. 1.

Calcium hydroxide apexification has been used over many decades as the treatment of choice for non-vital immature incisors where it has been essential to obtain a root end barrier in order to facilitate the placement of a root filling. However, it can be seen from the review that evidence for this technique cannot be deduced from well conducted RCTs.

Therefore the level of recommendation for Ca(OH)_2_ apexification is = C/D.

### Detrimental effects of prolonged dressing of root canal with calcium hydroxide

It has been suggested that due to its highly alkaline pH, Ca(OH)_2_ can cause desiccation of dentinal proteins thereby leading to the weakening of the tooth structure and predisposing these teeth to fractures. Prolonged dressing of the immature tooth with non-setting Ca(OH)_2_ has been shown to result in a reduction in the fracture strength of dentine. A retrospective study of luxated non-vital maxillary incisors treated with Ca(OH)_2_ in the root canal found that the frequency of cervical fracture was higher in these teeth (Al-Jundi 2004; Cvek 1992).

Level of Evidence = 2+.

In the last decade there have been a number of laboratory studies that have also shown a significant reduction of resistance to fracture of teeth following prolonged use of Ca(OH)_2_. These are: 

Andreasen et al. 2002.

Level of Evidence = 2++

Doyon et al. 2005; Rosenberg et al. 2007; Twati et al. 2009b.

Level of Evidence = 3.

### Recommended best practice based on the clinical experience of the guideline development group

In view of these findings clinicians should consider discarding the traditional approach of using prolonged dressing of root canals with Ca(OH)_2_ to achieve apexification and consider alternative methods of managing these teeth.

### Use of mineral trioxide aggregate (MTA)

Mineral trioxide aggregate (MTA) first received Food and Drug Administration of the USA (FDA) approval in 1998. It was later used in achieving an apical barrier in non-vital immature teeth. This treatment can be completed in one or two visits depending on the MTA used, thereby reducing the time needed for completion of treatment and restoring the tooth.

MTA is a powder that consists of fine hydrophilic particles that set in the presence of moisture. Hydration of the powder results in a colloidal gel with a pH of 12.5 that solidifies to a hard structure. MTA is available as grey or white and is made mainly of tricalcium silicate, dicalcium silicate, tricalcium aluminate, calcium sulphate dehydrate and bismuth oxide.

There are many favourable characteristics of MTA that indicates its use for managing non-vital immature teeth, these include:

- Reduced number of visits for patients.
- Good biocompatibility.
- Prevents microleakage.
- Induction of odontoblasts, cementoblasts and hard tissue barrier.
- Capacity to set in a moist environment.
- Radiopacity that is slightly greater than dentine.
- Low solubility.
- Setting time of 3–4 h.
- A pH of 12.5 after setting which is said to impart antimicrobial properties.
- Compressive strength after setting is 70 Mpa.
- Ability to create an apical stop that allows the tooth to be filled immediately.
- Potential for fracture of thin roots could be reduced as a bonded core can be placed immediately within the root canal.

MTA can be used to physically create a barrier at the root end thereby allowing the root canal obturation to be carried out in the same or the next visit. The following procedure is currently recommended:

1. Clean root canal system and dress with calcium hydroxide paste for at least 1 week.
2. Mix MTA immediately before its use.

Powder: sterile water (3:1).

3. Carry mix in a small amalgam carrier or MTA applicator.
4. Lightly condense the MTA with a plugger or back end of paper points.
5. Create a 3–4 mm apical plug and check radiographically.
6. Place a moist cotton pellet in the root canal and wait for 20-30 min or until next visit.
7. Obturate the root canal using thermoplasticised gutta percha or another obturation technique.
8. The coronal portion of the tooth is then restored and reinforced to prevent fractures.

In reviewing the evidence for MTA use it became very clear that the current available evidence does not meet the strict criteria set out by Cochrane collaboration. Most studies are in the form of case reports, case control/cohort or retrospective evaluations of cases (Table 2). However in our opinion these substantial numbers of studies supporting the use of MTA should not be overlooked. Conducting a prospective RCT on treatment outcome comparing Ca(OH)_2_ with MTA for managing non-vital immature permanent incisor teeth with an appropriate follow-up period is not only difficult but also expensive to undertake. Despite all the limitations in the reported studies, most if not all have demonstrated excellent clinical outcomes for non-vital immature teeth where MTA was used to create an apical plug, followed by root canal obturation. This is also supported by a recent systematic review and meta-analysis (Nicoloso et al. 2016) which concluded that MTA apexification appears to produce overall better clinical and radiographic success rates among endodontic treatment available in immature necrotic permanent teeth.

**Table 2:** Characteristics of clinical studies evaluating the use of MTA to facilitate obturation of root canal

Author	Level of evidence	n (Teeth)	Drop out rate	Age (years)	Follow-up (months)	Intervention	Out comes success
Lindeboom et al. (2005)	1-	100	NR	17–64	Follow-up after obturation 12m	MTA and IRM Apexification	MTA scored 92% success after one year BUT surgically treated
El-Meligy and Avery (2006)	2	30	NR	6–12	Follow-up after obturation 12m	Ca(OH)_2_ and MTA Apexification	100% MTA 87% Ca(OH)_2_
Pradhan et al. (2006)	2	Exp = 10 Cont = 10	NR	8–15	No follow-up	Ca(OH)_2_ and MTA Apexification	100%
Simon et al. (2007)	2	57 11GMTA 46WMTA	NR	Mean 18	Every 6 m for 24m & thereafter every 12 m for 48m	WMTA and GMTA Apexification	81% with minimum 1 year follow-up
Pace et al. (2007)	3	11	NR	11–32	1–2 yrs	Ca(OH)_2_/MTA Apexification	10 out of 11 = 90.9%
Moore et al. (2011)	1-	22	0	7–12	Follow-up after obturation for 12m & 18m	White MTA ProRoot(^®^) or white MTA Angelus MTA Apexification	Clinical success rate was 95.5% ProRoot Group: Absolute: 81.8% Relative: 90.9% Angelus MTA Group: Absolute: 100% Combined Groups: Absolute: 90.9% Relative: 95.5%
Damle et al. (2012)	1-	30	0	8–12	12m	Ca(OH)_2_ and MTA Apexification	Clinical and radiographic: MTA: 100% Ca(OH)_2_: 93.3%
Damle et al. (2012)	1-	30	3	6–18	12m	Ca(OH)_2_ and MTA Apexification	Clinical and radiographic calcific apical barrier: MTA: 82.4% Ca(OH)_2_: 50%

*Cont* Control; *Exp* Experimental; *GMTA* Grey mineral trioxide aggregate; *WMTA* White mineral trioxide aggregate; *NR* Not reported; *m* Months; *n* Number; *Ca(OH)*_*2*_ Calcium hydroxide

### Recommended best practice based on the available evidence

In view of these findings clinicians should consider using MTA routinely as a method for creating an apical barrier to allow root canal obturation to be carried out.

The level of recommendation for MTA = C.

### Disadvantages of MTA

Two potential problems have been reported with the use of MTA.

1. Discolouration of the crown (Adamidou 2010) leading to poor aesthetics in the long term.

Level of Evidence = 3.

2. It contributes to an increased brittleness of dentine and decrease in the fracture resistance of the tooth (Twati et al. 2011).

Level of Evidence = 3.

### Reinforcement of the coronal portion after endodontic management

It is important to reinforce the coronal portion of a tooth at the time of final restoration in order to increase the fracture resistance of endodontically managed immature teeth. There is a high frequency of coronal fractures reported for such teeth. (Cvek 1992).

Level of Evidence = 2+.

There is some evidence that fibre posts might be superior to other forms of restorations. (Bateman et al. 2003; Al-Ansari 2007).

Level of Evidence = 1+.

### Coronal seal

It is important to create a leak-proof coronal seal in order to prevent reinfection of the root canal with microorganisms as there is some evidence that coronal leakage contributes to the failure of endodontic treatment. (Quality guidelines for endodontic treatment 2006).

Level of Evidence = 3/4.

### Biological methods. The regenerative/revitalisation endodontic technique

In the last few years there seems to have been a paradigm shift in the way it is proposed to manage teeth with incomplete root development that have become non-vital as a result of trauma, caries or developmental anomalies such as dens-in-dente. The new way of thinking seems to have been prompted by the limitations of the use of Ca(OH)_2_ or MTA. Both of these methods allow root canal obturation to be performed through generating a physical barrier against which the root filling can be condensed. However, neither of these methods contributes to any qualitative or quantitative improvement in root dimensions. Rather the evidence reviewed above suggests that both methods can have a detrimental effect on dentine and might make the root more prone to fractures, in particular with the prolonged use of Ca(OH)_2_. If any further deposition of dentine or cementum is to be achieved, in order to provide a qualitative improvement of root structure, then vital tissue has to be generated, as only cellular activity can result in any such tissue being deposited. Recently there has been an attempt to re-establish the blood supply in those teeth, which have already become non-vital and the technique is commonly known as the Regenerative or Revitalisation Endodontic Therapy or technique (RET).

### Rationale

Through the repopulation with vital tissue of the root canal space, the RET technique aims to promote continued root development and/or thickening of the dentinal walls, thereby improving the long-term prognosis of the tooth.

The technique is based on the following prerequisites:

1. Presence of stem cells.
2. Complete disinfection of the root canals.
3. Provision of a scaffold within a root canal.
4. Provision of a signal to the stem cells in order that they can differentiate.

### Harnessing the potential of stem cells in the apical area

There are several sources of stem cells in the oral cavity (Hargreaves et al. 2013) with some researchers implying that stem cells of the apical papilla (SCAP) have a major role in regeneration techniques (Huang et al. 2008). Recently it has been shown that stem cells exist in the apical area of incomplete roots in children and adolescents (Sonoyama et al. 2008). The entity in which these cells exist has been called as stem cells of the apical papilla (SCAP). Sonoyama and co-workers (2008) demonstrated that isolated SCAP grown in cultures have the ability to undergo dentinogenic differentiation when stimulated with dexamethasone supplemented with L-ascorbate-2 phosphate and inorganic phosphate. SCAP cells have also been shown to be capable of differentiating into functional dentinogenic cells in vivo, using implantation techniques in animal experimental models. In summary SCAP have been shown to be similar to dental pulp progenitor cells and therefore, if their potential can be harnessed they could be induced to differentiate into dental pulp cells. Stem cell population growth into the root canal system is achieved mainly through induction of bleeding from the periapical area, which has been achieved in 77% of the work published until May 2014 (Kontakiotis et al. 2015). This has been supported by the work of Lovelace et al. (2011)who showed a 400-600 fold increase in mesenchymal stem cell markers in blood collected from root canals in comparison to those levels found in systemic blood samples.

Following this understanding a technique has been proposed which could harness the potential of the SCAP cells leading to repopulation of the root canal space with vital tissue. This technique has been referred to as revitalisation, revascularisation, repopulation, regeneration or even maturogenesis (Wigler et al. 2013). The exact nature of the tissue repopulating the root canal system is still unclear with histological studies reporting desirable tissues such as fibroblasts, blood vessels and collagen and undesirable tissues such as cementoblasts and osteoblasts (Wigler et al. 2013).

### Achieving disinfection of the root canal

The use of sodium hypochlorite, with concentrations of 1–6%, has been used either as the only irrigant (65% of studies) or in combination with other irrigants in 97% of RET studies published before May 2014 (Kontakiotis et al. 2015). This irrigant has been shown to be a potent antimicrobial material that dissolves organic matter (Martin et al. 2014).

Some laboratory studies have investigated the effect of sodium hypochlorite on stem cells. Martin et al. (2014) assessed the effect of different sodium hypochlorite concentrations (0.5, 1.5, 3 and 6%) followed by either 17% EDTA or normal saline and reported negative effects of high concentration of sodium hypochlorite on the survival and differentiation of SCAP. They recommended the use of 1.5% sodium hypochlorite followed by 17% EDTA. The use of EDTA following irrigation with sodium hypochlorite is now widely recommended (Wigler et al. 2013). Trevino et al. (2011) assessed the effect of different combinations of irrigants on SCAP and reported the best outcome, in terms of cell survival, was following irrigation with only 17% EDTA. Therefore the use of 1.5% sodium hypochlorite followed by 17% EDTA is currently the recommended irrigation system in RET and should be employed in future studies.

The use of an antibiotic paste had been reported in 80% of studies published (Kontakiotis et al. 2015). A tri-antibiotic paste containing 100 mg Metronidazole, 100 mg Minocycline and 100 mg Ciprofloxacin has been shown to have a sufficient bactericidal efficacy and potency to eradicate bacteria from the infected dentine of root canals (Hoshino et al. 1996). Recently, Minocycline has been eliminated from the mixture due to its potential to discolour the tooth (Kim et al. 2010) which was further supported by recent work conducted showing similar antimicrobial effects of the tri-antibiotic and bi-antibiotic pastes (Twati et al. 2011).

Achieving a hermetical coronal seal is also crucial in maintaining a sterile root canal environment. The use of MTA in achieving a hermetic coronal seal, hence preventing future contamination, had been associated with crown discolouration. The most commercially available MTA contains agents used to enhance its radio-opacity, such as bismuth oxide, which is known to cause discolouration of teeth. There are currently several types of contemporary materials with similar biocompatibility and biomineralisation that are recently gaining popularity as suitable materials as a viable replacement for MTA. These include materials e.g. Biodentine^®^, EndoSequence^®^ Root Repair Material and Portland cement (Lenherr et al. 2012; Nazzal et al. 2015). In vitro and in vivo studies have used bioceramics to demonstrate antibacterial effects (Elshamy et al. 2016), biocompatibility to pulp tissue and induction of dental pulp cells proliferation and reparative dentine bridge formation (Liu et al. 2015), whilst producing significantly less discolouration. It is certain that the recommendations for coronal seal material will change as more information on the suitability of these materials become available.

### Providing a scaffold and signal for stem cells to differentiate

There are an increasing number of commercial scaffolds available for tissue engineering in the medical field but these are too expensive for use in dental practice. A biological scaffold is required within the root canal, which would serve two purposes. Firstly it would provide a matrix into which the cells from SCAP could differentiate. Secondly it should act as a scaffold rich in growth and differentiation factors that are essential to aid with the in-growth of viable tissue into the pulpal space. Currently a blood clot is considered as a favourable scaffold for this technique. The use of blood clot as a scaffold has been used in 75% of RET protocols published before May 2014 (Kontakiotis et al. 2015). Various other scaffolds have been suggested and/or used, such as platelet rich plasma (PRP) and platelet rich fibrin (REF) but these have not shown any added advantage over the use of a blood clot.

### Recommended clinical technique

Despite the recently published American Association of Endodontics RET protocol, different modifications of RET have been used by researchers (Kontakiotis et al. 2015).

The outline of the technique proposed in general is as follows:

- All procedures are carried out under administration of local analgesia and rubber dam isolation.
- Pulpal extirpation and copious chemical irrigation of root canals with a mild disinfectant such as 1.5% sodium hypochlorite is performed.
- Minimal or no filing to the root canal is carried out to prevent further weakening of the existing dentinal walls.
- The tooth is then dried and the root canal filled with the double mixture antibiotic paste (Metronidazole and Ciprofloxacin, 1:1). The use of triple antibiotic pastes that contain minocycline or antibiotics belonging to the tetracycline group should be avoided due to the discolouring effect. Caution should be exercised when using any antibiotic paste, to ensure that its application is below the cervical margins in order to prevent discolouration of the crown. Alternatively, clinicians can consider using non-setting Ca(OH)_2_ for 2–3 weeks in order to achieve root canal disinfection.
- The tooth is sealed temporarily and a review is scheduled after 2-4 weeks depending on the degree of signs and symptoms of infection. It is essential that disinfection of the root canal is carried out until there is no evidence of purulent discharge, sinus tract or infection, and the disinfection process should be repeated if the root canal is still not infection-free.
- At the next appointment, the canals should be irrigated with copious amounts of normal saline followed by copious amounts of 17% EDTA. The canal is then dried with paper points after which a sterile 23-gauge needle or a long endodontic instrument such as a file or a finger spreader is pushed 2 mm beyond the working length, beyond the confines of the root canal into the periapical tissues, to intentionally induce bleeding into the root canal. The bleeding is allowed to fill the root canal. The use of local analgesia will be required and it is preferable to use one without a vasoconstrictor to facilitate induction of bleeding into the canal space.
- When frank bleeding is evident at the cervical portion of the root canal, a cotton pellet is then inserted 3–4 mm into canal below the cervical margins and held there for about 7–10 min to allow formation of a blood clot. This blood clot acts as a scaffold rich in growth and differentiation factors that are essential to aid in the in-growth of viable tissue into the pulpal space and in wound healing processes.
- The access is sealed with a material such as Portland cement/MTA, followed by glass ionomer cements and or composite resin to ensure an excellent coronal seal, extending about 4 mm into the coronal portion of the root canal. The use of resorbable matrices such as CollaPlug^TM^, Collacote^TM^, CollaTapeT^M^ over the blood clot as suggested by the AAE (American Association of Endodontics 2016) could be considered, to reduce the risk of discolouration of the crown.
- Periapical radiographs are then taken as a baseline record. This step is essential for comparison with future 6-monthly radiographs to ascertain continued root development and thus success of the treatment.

### Review of current evidence

 The original idea as proposed by Nygaard-Ostby (1961) regained popularity since its use by Banchs and Trope (2004). In the last few years, several studies have been published including a few RCTs comparing different types of scaffolds or RET against other non-vital immature teeth management techniques such as apexification or MTA apical plug technique. An analysis of the studies that are relevant is given in Table 3.

**Table 3:** Characteristics of clinical studies evaluating the use of regenerative endodontic technique (RET)

Study	Level of evidence	Aetiology	Age (years±SD)	Groups T = treatment C = control	Follow-up (months±SD)	Periapical healing	Continued root development	Dentinal thickening of walls	Apical closure
Bose et al. (2009)	2-	Variable = 88	–	T1 = RET TAP SNR T2 = RET Ca(OH)_2_ SNR T3 = RET FC SNR C1 = MTA C2 = NSRCT	0 to >36	–	RET TAP and RET Ca(OH)_2_ produced significantly greater increases than MTA or NSRCT	RET TAP produced significantly greater differences than RET Ca(OH)_2_ or RET FC	–
Jadhav et al. (2012)	1-	Trauma = 20	15–28	T = RET TAP PRP + BC (n = 10) C = RET TAP BC (n = 10)	12	T= 70% ++ C= 40% ++ 50% +++	T= 10% + 50% ++ 40% +++ C= 40% + 60% ++	T= 20% + 50% ++ 30% +++ C= 30% + 70% ++	T= 30% ++ 20% +++ C= 30% ++ 70% +++
Jeeruphan et al. (2012)	2+	Caries = 5 Anomaly = 20 Trauma = 36	T:12.9 ± 5 C1: 14.6 ± 6 C2: 10.5 ± 3.8	T = RET TAP BC + Collaplug (n = 20) C1 = MTA(n = 19) C2 = Ca(OH)_2_ (n = 22)	T = 21 ± 12 C1 = 14 ± 8 C2 = 27 ± 30	T = 80% (16/20) C1 = 68.42% (13/19) C2 = 77.3% (17/22)	T = 14.9% C1 = 6.1% C2 = 0.4%	T = 28.2% C1 = 0% C2 = 1.52%	–
Alobaid et al. (2014)	2+	Trauma = 24 Caries = 4 Anomaly = 3	T = 8.8 ± 1.6 C = 9.8 ± 2.0	T = RET^b^ BC (n = 19) C = MTA (n = 12)	T = 14 ± 8.5 C = 21.8 ± 12	–	T = 0%^a^ C = 12.5% (1/8)^a^	T = 20% (3/15)^a^ C = 0%^a^	NR
Nagata et al. (2014)	1-	Trauma = 23	7–17	T1 = RET TAP BC (n = 12) T2 = CaOH_2_ (n = 11)	1–19	T1 = 100% T2 = 80%	T1 = 41.7% T2 = 27.3%	T1 = 41.7% T2 = 45.4%	T1 = 66.7% T2 = 54.5%
Nagy et al. (2014)	1-	Trauma = 36	9–13	T1 = RET TAPD BC (n = 12) T2 = RET TAPD FGF (n = 12) C = TAPD MTA (n = 12)	3–18	T1 = 100% T2 = 90% C = 80%	–	–	T1 = 100% T2 = 90% C = 80%
Bezgin et al. (2015)	1-	Trauma = 14 Caries = 6	7–13	T = RET TAPC PRP (n = 10) C = RET TAPC BC (n = 10)	18	T = 100% (7/7) C = 88.9% (8/9)	–	–	T = 70%; (7/10) C = 60% (6/10)
Narang et al. (2015)	1-	NR	<20	C = MTA (n = 5)	6 and 12	C = 40% +++	C = 0%	C = 0%	C = 0%
						60% ++			
				T1 = RET TAP BC (n = 5)		T1 = 40% +	T1 = 60%+	T1 = 50% +	T1 = 33.3% ++
						60% ++	40% ++	50% ++	66.6% +++
				T2 = RET TAP PRF (n = 5)		T2 = 98% +++	T2 = 100%+++	T2 = 40% ++	T2 = 60%+
						2% ++		60% +++	40% ++
				T3 = RET TAP PRP collagen (n = 5)		T4 = 20% +	T3 = 60%+	T3 = 80% +	T3 = 40%+
						80% ++	40% ++	20% ++	60% ++

+ Satisfactory, ++ good, +++ excellent, *T* test group, *C* control group, *RET* regenerative endodontic technique, *BC* Blood Clot, *PRP* platelet rich plasma, *PRF* platelet rich fibrin, *TAB* tri antibiotic paste (ciprofloxacin, minocycline, metronidazole), *TAPC* tri antibiotic paste (Ciprofloxacin, minocycline, cephaclor), *TABD* tri antibiotic paste (ciprofloxacin, doxycycline, metronidazole), *Ca(OH)*_*2*_ calcium hydroxide, *FC* ferric sulphate, *MTA* mineral trioxide aggregate, *NSRCT* conventional RCT with gutta purcha, *GP* gutta purcha only, *FGF* blood clot and an injectable hydrogel scaffold impregnated with basic fibroblast growth factor, *UK* unknown scaffold, *NR* not reported

^a^Results when a 20% or more increase in root dimension is deemed clinically significant, ^b^ varying intracanal medicament

### Recommended best practice based on the clinical experience of the guideline development group

At present there is insufficient evidence available for this technique to be recommended for use routinely by clinicians for the management of non-vital immature teeth in children. However, it is suggested that clinicians should give due consideration to the use of this method especially in cases where the root development is very immature and even the use of MTA is unlikely to improve the prognosis of the tooth.

The level of recommendation for RET = D.

### Recommendations of best practice, based on the available evidence, for the management of non-vital anterior teeth with incomplete root development

1. There seems to be sufficient evidence to make a recommendation that the prolonged use of Ca(OH)_2_ in root canals of non-vital immature teeth should be avoided. Therefore this use of Ca(OH)_2_ for the traditional apexification technique is no longer advocated as the treatment of choice for such teeth.
2. For non-vital anterior teeth with incomplete root development, and/or a wide open apex it is recommended to use Ca(OH)_2_ for a short period of time to achieve disinfection. This should be followed by the application of MTA to create a barrier, obturation of root canal space with gutta percha and finally the creation of a good coronal seal to prevent re-infection of the root canal space. **This should be the treatment of choice based on the current systematic review.**
3. Clinicians should consider using the RET in cases where the root development is very incomplete with insufficient amount of dentine, and where it is considered that the tooth has a hopeless prognosis even with application of MTA. In these cases it would be advantageous to gain some deposition of hard tissues through a regenerative approach. However, this is based on weak evidence.
